# Emerging role of different DNA methyltransferases in the pathogenesis of cancer

**DOI:** 10.3389/fphar.2022.958146

**Published:** 2022-08-25

**Authors:** Pengcheng Liu, Fan Yang, Lizhi Zhang, Ying Hu, Bangjie Chen, Jianpeng Wang, Lei Su, Mingyue Wu, Wenjian Chen

**Affiliations:** ^1^ Department of Human Resources, The First Affiliated Hospital of Anhui Medical University, Hefei, China; ^2^ The First Clinical Medical College, Anhui Medical University, Hefei, China; ^3^ Inflammation and Immune Mediated Diseases Laboratory of Anhui Province, School of Pharmacy, Anhui Medical University, Hefei, China; ^4^ Department of Oncology, The First Affiliated Hospital of Anhui Medical University, Hefei, China; ^5^ Department of Orthopaedics, Anhui Provincial Children’s Hospital, Hefei, China

**Keywords:** DNMT, cancer, cellular signaling pathways, epigenetics, anticancer therapy

## Abstract

DNA methylation is one of the most essential epigenetic mechanisms to regulate gene expression. DNA methyltransferases (DNMTs) play a vital role in DNA methylation in the genome. In mammals, DNMTs act with some elements to regulate the dynamic DNA methylation patterns of embryonic and adult cells. Conversely, the aberrant function of DNMTs is frequently the hallmark in judging cancer, including total hypomethylation and partial hypermethylation of tumor suppressor genes (TSGs), which improve the malignancy of tumors, aggravate the ailment for patients, and significantly exacerbate the difficulty of cancer therapy. Since DNA methylation is reversible, currently, DNMTs are viewed as an important epigenetic target for drug development. However, the impression of DNMTs on cancers is still controversial, and therapeutic methods targeting DNMTs remain under exploration. This review mainly summarizes the relationship between the main DNMTs and cancers as well as regulatory mechanisms and clinical applications of DNMTs in cancer and highlights several forthcoming strategies for targeting DNMTs.

## 1 Introduction

DNA methylation and histone modification are both epigenetic modifications; that is, mammalian cells have unchanged DNA sequences and genetically altered gene expression. Both are mediated by the interaction between DNA methyltransferases (DNMTs) and histone deacetylases (Pan et al., 2019). Under normal circumstances, DNA hypermethylation is mainly regulated by the DNMT family, including DNMT1, DNMT3A, and DNMT3B. Promoter hypermethylation and TSG deacetylation mediated by DNMTs caused gene transcriptional silencing, which suggested a major role for DNMTs in tumorigenesis. For example, there is a positive correlation between DNMT overexpression and induction in hepatocellular carcinoma (HCC) (Sanaei et al., 2018). Some results also showed that DNMT1 regulated epithelial–mesenchymal transition and cancer stem cells to promote prostate cancer (PCa) metastasis (Lee et al., 2016).

Cancer, a puzzling and frightening disease or set of diseases, is the leading cause of clinical, social, and economic burden (Kiisholts et al., 2021). Currently, cancer has become the second reason of death worldwide after ischemic heart disease and is projected to become the first in 2060 (Hu et al., 2022). According to the latest GLOBOCAN 2020 estimates, a total of 19.3 million new cases were diagnosed in that year, among which were the common cancers: over 2.2 million cases each of lung and breast cancer (BC) and 1.41 million cases of PCa (Sung et al., 2021). It is well known that aberrant activation of oncogenes or inactivation of tumor suppressor genes (TSGs) is considered to induce the deregulation of critical signaling pathways governing cell proliferation and apoptosis, causing the malignant transformation of stem cells and carcinogenesis (Zhou et al., 2016). Therefore, it is imperative to explore novel therapies for cancer treatment at the genetic level. Cancer, habitually believed as a hereditary disease, is now regarded as resulting from epigenetic irregularities associated with genetic changes. However, the lack of profound research on epigenetics of cancer is a major obstacle to curing cancer nowadays. To carry out effective anticancer therapy, understanding the role of methylation on oncogenes and TSGs may be a breakthrough.

In this review, we generalized the functions and regulatory roles of DNMTs among cancers. Also, their potential clinical applications in these cancers will be discussed. Therefore, it can be tentatively concluded that DNMTs have the potential as diagnostic and prognostic markers for cancer.

## 2 Overview of DNMTs

**FIGURE 1:**
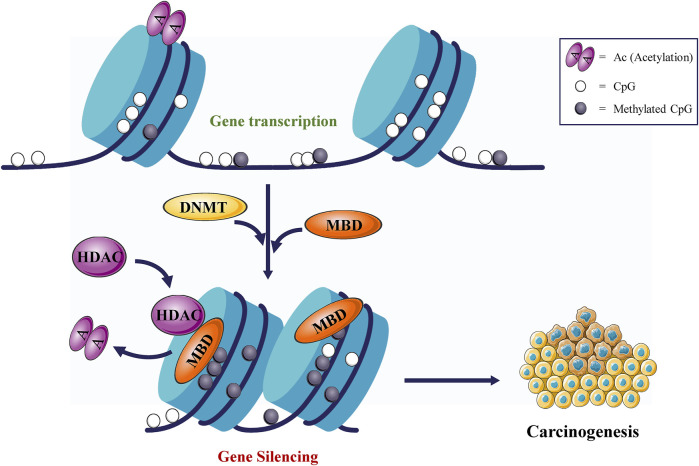
DNMTs promote tumorigenesis. DNMTs cause gene silencing and promote tumorigenesis.

DNA methylation refers to the process of obtaining one methyl (CH3) group in a covalently bound manner, which is a chemical modification process of S-adenosylmethionine (SAM) as a CH3 donor under the catalysis of DNMTs at a specific base on DNA sequence (Li et al., 2021). During the process, cytosine (C) is the most common base that can be methylated. It is worth noting that the methylation of the C site mainly occurs on the CpG sequence. Among mammals, parts of the CpG dinucleotides are dispersed in the genome, while others appear in dense clusters named CpG islands. CpG islands are generally unmethylated, which are usually located in gene promoter regions that modulate transcriptional initiation and repression (Erdmann et al., 2015). The hypermethylation of CpG sites in enhancers or promoters usually resulted to transcriptional silencing, while the hypomethylation of CpG sites in genomes usually leads to the activation of gene expression (Morgan et al., 2018) (Figure 1). Thus, methylation often regulates gene expression through the transcription of genes. It was mentioned earlier that DNA methylation is mainly dependent on DNMTs, whereas there are five members in the DNMT family: DNMT1, DNMT2, DNMT3A, DNMT3B, and DNMT3L (Bestor et al., 1988). DNMT1 is involved in the holding of sequence methylation during cellular proliferation (Jeltsch and Jurkowska, 2014), which is necessary for the faithful maintenance of DNA methylation patterns, as well as abnormal silencing of TSGs in cancer cells (Chen et al., 2007). Nonetheless, DNMT2 acts as an RNA methyltransferase modifying the 38th cytosine residue in the anticodon loop of certain tRNAs (Ashapkin et al., 2016). Moreover, the major role of DNMT3A and DNMT3B is *de novo* methylation (Shen and Laird, 2013), while DNMT3L belongs to the DNMT3 family but lacks methyltransferases activity (Liao et al., 2012). However, DNMT3L can interact with DNMT3A/B to facilitate *de novo* DNA methylation (Yu Y. C. et al., 2020). DNMT3A has two distinct isoforms, while DNMT3B has more than 30 isoforms. Their common feature is that both have a catalytically active and structurally conserved C-terminal domain, responsible for binding the SAM cofactor and targeting cytosine. That is why they can therefore catalyze the transfer of a CH3 group from SAM to the C5 position of cytosine to form 5-methylcytosine (Reichard and Puga, 2010). Taken together, DNMT1 is involved in the maintenance of sequence methylation, whereas the main role of DNMT3A and DNMT3B is *de novo* methylation. They can also be involved in the maintenance of methylation.

Additionally, these three DNMTs are frequently found to be overexpressed in several cancer tissues and cell lines. Combined with the reversibility of DNA methylation, DNMTs are known as important epigenetic targets for drug development (Zhou et al., 2018). An increasing number of studies have observed methylation alterations at the CpG sites in tumor cells, which represent aberrant DNA methylation that may be present in cancer and many nonmalignant diseases. Fortunately, these changes showed concordance with the differentiation caused by DNA methylation at promoters, enhancers, gene bodies, and sites that control higher-order chromatin structure. Thus, aberrant changes in DNA methylation may be a breakthrough in the study of cancer formation and tumor progression. In addition to cancer, aberrant DNA methylation is associated with some neurological diseases, immune disorders, atherosclerosis, and osteoporosis (Zhou et al., 2018; Ehrlich, 2019). Therefore, understanding the molecular roles of aberrant DNMTs will lay theoretical foundations for clinical research and the development of new drugs.

## 3 Overview of cancer

Cancer has become a major global public health problem (Siegel et al., 2019). Some hallmarks constitute an organizing principle for rationalizing the complexities of neoplastic disease, including maintaining cancer-promoting inflammation, proliferative signaling, escaping growth suppressors, evading immune destruction, fighting back cell death, achieving replicative immortality, causing angiogenesis, inducing invasion and metastasis, genome instability, and reprogramming of energy metabolism (Hanahan and Weinberg, 2011; Hanahan, 2022). According to the WHO statistics, cancer caused the greatest burden worldwide with up to 244.6 million disability-adjusted life years (DALYs), of which 137.4 million and 107.1 million DALYs are ,respectively, for men and women (Mattiuzzi and Lippi, 2019). In recent years, both the incidence and mortality of cancer have been increasing, and it has become the leading cause of death in China and America (Siegel et al., 2022; Xia et al., 2022). In addition, analyzing the statistics of China in 2020, the most common cancers in China were mainly lung cancer (accounting for 17.9%, about 0.82 million new cases), colon cancer (accounting for 12.2%, about 0.56 million new cases), and stomach cancer (accounting for 10.5%, about 0.48 million new cases) (Sung et al., 2021).

Cancer is generally considered to be a disease driven by progressive genetic abnormalities, including TSG mutations, oncogene mutations, and chromosomal abnormalities (Litwin et al., 2017). Meanwhile, cancer is also caused by epigenetic alterations, and the reversibility of epigenetic alterations allows the effect on biological traits to be achieved by removing epigenetic alterations under the condition of certain factors (Yang Y. et al., 2017). From this, it can be seen that the rapid development of epigenetics has revealed complex genetic phenomena not only at the molecular level but has raised hope for conquering diseases, especially in the field of cancer. The regulation of DNA methylation, histone modifications, and noncoding RNAs turn into candidates for epigenetic therapy. Among them, disruption of DNA methylation is demonstrated to be closely related to multiple types of cancer, especially related to TSGs that play important roles in tumor development and progression (Liang et al., 2019). Owing to the fact that DNA methylation happened in the early stages of cancer, the transcriptional status of these genes can be regulated by the inhibition of DNMT activity. Thus, these genes not only provide valuable biomarkers but also potential targets for therapy. For instance, DNA demethylating agents, such as the nucleosides 5-azacytidine and decitabine, have been successfully used at low doses to treat leukemia and myelodysplastic syndromes (MDSs) (Li et al., 2013). In summary, the application of target-specific DNMT inhibitors in cancer therapy is of great potential, but its role in treating solid tumors remains unclear.

## 4 The functional role of various types DNMTs in cancer

Carcinogenesis of normal cells is mostly derived from functional changes in genetic materials caused by oncogenic factors (Rider and Cramer, 2015). Aberrant methylation patterns have been frequently found in various types of tumors by many researchers, indicating that the occurrence and development of cancers were accompanied by changes in DNMT, such as some hematological malignancies. Recently, some studies have shown that the great difference in DNMT1 expression is one of the bases for its possible use as a potential biomarker for demethylation therapy (Honeywell et al., 2018). In addition, DNMT3A is found to be mutated at a high rate in acute myeloid leukemia (AML) and associated with poor prognosis. DNMT3B is also often proved to be overexpressed in tumor cells, for instance, it plays a cancer-promoting role in human melanoma. Similarly, the lack of DNMT3B significantly inhibits melanoma formation in the BRAF/phosphatase and tensin homolog (PTEN) mouse melanoma model (Zhang et al., 2020). These findings have suggested that different DNMTs played vital roles in carcinogenesis. In this review, we mainly summarize the roles of DNMT1, DNMT3A, and DNMT3B in cancer, hoping to shed new light on the treatment of cancer (Tables 1, 2).

**TABLE 1:** DNMTs and their expression in various cancers.

DNMT	Cancer types	Cell/tissue	Expression in cancers	Ref.(PMID)
DNMT1	Lung cancer	HCC827	+^a^	32211850
	Gastric cancer	SGC7901, BGC-823, MGC-803, NCI-N87	+	33962804
	Breast cancer	MCF-7, T47D, MDA-MB-231, BT549, BT474, SKBR3, Hs578T	+	31296961
	Pancreatic cancer	PL45, PSN1, Panc1, BxPC3, AxPC1, SW1990, Capan1	+	35182659
				32504382
	Prostate cancer	PC3, DU145	+	27659015
	Colorectal cancer	HT-29, HCT116, SW620, LoVo	+	31154022
DNMT3A	Acute myeloid leukemia	Serum	+^c^ (87.5%)	34100902
	Vulvar squamous cell carcinoma	Epithelium of vulva	+(44%)	27623253
	Gastric cancer	BGC-823, AGS	+	33931579
	Lung cancer	A549, NCI-H1395, NCI-H226, NCI-H460, NCI-H520	+	27237029
	Colorectal cancer	HCT116, RKO, SW480, SW620	+	35414793
DNMT3B	Endometrial cancer	ISK, KLE	+	34175897
	Colon cancer	HT29, HCT116	+	30540937
	Lymphoma	RAJI, Daudi, Akata, JeKo-1, JVM-2, JURKAT, REH, JY	**-** ^b^	22133874
	Breast cancer	MDA-MB-231, MCF-7	+	30851420

a “+” indicates an increase in expression

b “-” indicates a decrease in expression

c The proportion of cases in which the reference mentions up- or downregulation of DNMT

**TABLE 2:** Downstream genes or proteins of DNMTs in cancers.

DNMT	Cancer types	Downstream genes or proteins	Ref.(PMID)
DNMT1	Lung cancer	Human mutL homolog 1(hMLH1), Human mutS homolog 2 (hMSH2)	32211850
	Gastric cancer	p53	33962804
	Breast cancer	Forkhead box O3A (FOXO3a)	31296961
	Pancreatic cancer	Homeobox B9 (HOXB9)	35182659
	Prostate cancer	Zinc finger E-box-binding homeobox2 (ZEB2), Kruppel like factor 4 (KLF4)	27659015
	Colorectal cancer	CDKN1A(p21)	31154022
DNMT3A	Acute myeloid leukemia	Cyclin-dependent kinase 1 (CDK1)	34067359
	Vulvar squamous cell carcinoma	Cyclin-dependent kinase inhibitor 2A (CDKN2A)	27623253
	Gastric cancer	ADAM metallopeptidase with thrombospondin type 1 motif 9 (ADAMTS9)	33931579
	Lung cancer	CD147	35132181
	Colorectal cancer	TIMP metallopeptidase inhibitor 2 (TIMP2)	35414793
DNMT3B	Endometrial cancer	Transcription factor 3 (TCF3)	34175897
	Colon cancer	Interferon regulatory factor 8 (IRF8)	30540937
	Lymphoma	Met proto-oncogene (MET)	33418509
	Breast cancer	C-X-C motif chemokine ligand 11 (CXCL11)	35390315

### 4.1 DNMT1 in cancer

DNMT1 gene, which is situated on chromosome 19p13.2, has a global protein-coding region of 4,851 bp and encodes approximately 183 kDa protein (Li and Tollefsbol, 2010). Moreover, DNMT1, which is able to maintain a DNA methylation pattern, is regarded to have a high affinity for hemimethylated DNA templates (Holliday et al., 2018). It is well-documented that DNMT1 is an oncoprotein in both solid and hematological malignancies (Pathania et al., 2015; Wong et al., 2019), and DNMT1 has been reported to exert oncogenic effects in multiple cancers, such as BC (Li H. et al., 2020; Zhu et al., 2022), lung cancer (Wu et al., 2020), colon cancer (Chen et al., 2019), and osteosarcoma. Therefore, researchers have been pushing to investigate the mechanism of DNMT1 in cancer, aiming to improve the effectiveness of comprehensive treatment.

Fortunately, Belinsky et al. (2003) used a mouse model containing an allele with the DNMT1 gene, disrupted to study the role of DNMT in cancer formation. In the process, they found that the decrease of DNMT1 caused a reduction in the occurrence of lung cancer resulting from tobacco tormogens, and DNMT activity was also suppressed in pneumocytes that could induce lung cancer. The results revealed that decrease in DNMT1 and histone deacetylase activities, that probably block epigenetically mediated gene silencing, might provide an emerging clinical strategy to help prevent lung cancer. In addition, compelling evidence suggests that human MutL Homolog 1 (hMLH1) and human MutS Homolog 2 (hMSH2) are closely associated with the development and drug resistance of cancer (Xing et al., 2019). Based on the previous study, Wu et al. (2020) found that DNMT1 could suppress the expressions of hMLH1 and hMSH2 *via* promoting their promoter methylation, thus promoting cell proliferation in epidermal growth factor receptor (EGFR)-mutated non-small-cell lung cancer (NSCLC). Moreover, EGFR has been a therapeutic target in human malignancies because of its frequent overexpression and overactivation, which is reflected in the results. Notably, microtubule-associated tumor suppressor 1 (MTUS1) has been recognized as a TSG in a variety of tumors (Sengupta et al., 2018). In a previous study of Parbin et al. (2019), treatment with DNMTs inhibitor resulted in both reduced promoter methylation accompanied by the enrichment of H3K9Ac and upregulated MTUS1 expression. Hence, they concluded that DNMT1 expression level was upregulated in NSCLC cell line A549, and there was an inverse correlation between DNMT1 and MTUS1 function.

To date, aberrant DNA methylation has been also shown to play an important role during carcinogenesis in pancreatic ductal adenocarcinoma (PDAC), with approximately 80% of cancer overexpressing the DNMT1 protein (Xu et al., 2010). For instance, Li et al. (2010) analyzed 20 pancreatic cancer (PC) cell lines and found that 16 of them had approximately 2–5 fold higher expression levels than the reference non-neoplastic cell lines. Additionally, it is well-documented that p53 is an important TSG and its inactivation is highly correlated with tumorigenesis (Duffy et al., 2022). On the basis of this, Godfrey JD et al. demonstrated that a large proportion of PDACs carried specific mutations in TP53, such as p53^R172H^, which imparted additional gain-of-function characteristics that promote metastasis. This study revealed that the DNMT1 expression level was all elevated from the PDAC models, the Kras p53^R172H^ tissues, and Kras p53^R172H^ cell line compared to the normal group (Godfrey et al., 2018). In this experiment, rat sarcoma (RAS) is the most regularly mutated oncogene in cancer, with Kirsten rat sarcoma (KRAS) becoming the most frequently mutated RAS isoform, accounting for over 80% of RAS mutations observed in human cancer (Liu et al., 2019). Apart from abovementioned studies, Wong et al. validated that DNMT1 plays oncogenic roles in inhibiting PDAC cell differentiation and promoting its proliferation, invasion, and migration, as well as in the induction of the self-renewal capacity of PDAC cancer stem cells *via* promoter hypermethylation of TSGs (Wong, 2020).

Etoh et al. found that DNMT1 could not be discovered in normal epithelium, but was discovered in most gastric cancers (GC). In addition, the hyperexpression of DNMT1 in GC tissues was greatly correlated to reduced E-cadherin expression, which indicated that an increase in the DNMT1 expression level would elevate the migration ability of the cancer cells (Etoh et al., 2004). LncRNAs have been reported to modulate the target genes *via* methylation modification, such as lncRNA SAMD12-AS1. Functionally, lncRNA SAMD12-AS1 could enhance the binding of DNMT1 to p53 and accelerate its degradation of p53, thereby inhibiting the occurrence and development of GC (Lu et al., 2021). Similarly, it has been demonstrated that the increased expression level of DNMT1 was regulated by tumor-associated macrophages (TAMs), while the upregulated DNMT1 in turn aggravated GC *via* tumor suppressor gelsolin, that mediated epigenetic repression (Wang H. C. et al., 2017).

Previous research has shown that antigen presentation mediated by major histocompatibility complex (MHC) was also recognized as one of the considerable modifiers of antitumor immunity and response to PD-1/L1 targeted therapy (Ebert et al., 2016). With this conclusion as a background, using immunohistochemistry, Luo et al. (2018) found that there were no remarkable changes in the total CD8^+^ T cell infiltrate after treating by guadecitabine (DMTi, DNA methyltransferase inhibitors). Instead, a significant increase was observed in the proportion of CD8^+^ T cells infiltrating into the tumor region compared to the stromal parenchyma (*p* = 0.024). In addition, further experiments uncovered that EpCam (epithelial cell adhesion molecule) is a type I transmembrane glycoprotein, which was originally identified as a tumor-associated antigen (Huang et al., 2018). MHC-I on EpCam^+^ tumor cells demonstrated an upregulation of MHC-I on tumor cells with DMTi. These data suggested that DNMT1 could affect MHC-I promoter methylation, and change the immune microenvironment. Additionally, G protein subunit alpha O1 (GNAO1), one of the members of Guanine nucleotide-binding protein (G protein), has been recognized to be a tumor suppressor protein whose deregulation can promote carcinogenesis. A study has demonstrated that DNMT1 mediated hypermethylated promoters silence the GNAO1 gene (Pei et al., 2013), leading to the development of HCC (Xu et al., 2018). Taken together, the abovementioned studies suggest that DNMT1 has cancer-promoting effects and served as the potential target in cancer treatment.

### 4.2 DNMT3A in cancer

The DNMT3A gene, located on chromosome 2, has a protein-coding region of 2,172 bp and encodes a protein of about 130 kDa (Xie et al., 1999). According to previous research, DNMT3A binding to DNA is mainly mediated by a loop from the target recognition domain (TRD), the catalytic loop, and the homodimeric interface of DNMT3A, which jointly establishes a continuous DNA-binding surface (Zhang et al., 2018). Typically, DNMT3A is thought to have a preference for unmethylated DNA, resulting in *de novo* methylation (Lakshminarasimhan and Liang, 2016). Data from several studies suggested that the two DNMT3A monomers can comethylate two adjacent CpG dinucleotides in one DNA-binding event (Ren et al., 2018). Among the DNMTs, mutations in DNMT3A have been reported to be most frequent in cancer. Moreover, research from several sources has identified that hyperexpression of DNMT3A was associated with malignant characteristics of vulvar squamous cell carcinomas (Leonard et al., 2016), pituitary adenomas (Ma et al., 2018), and colorectal cancer (CRC) (Zhang et al., 2022), such as high invasion and migration.

Interestingly, Liu et al. and Husni et al. (Husni et al., 2016) pointed out different views that both the overexpression and deletion of DNMT3A favor the development of lung cancer. Up till now, increasing evidences have indicated that miRNAs took roles in cancer progression and participated in the process of cancer (He et al., 2020), consisting of miR-708-5P and miR-101. In the experiments of Liu et al. (2018), protein translation of DNMT3A was suppressed by miR-708-5P that functions as a protective factor, which reduced the tumorigenicity of NSCLC cells. In addition, miR-101 is frequently silenced in human cancers, more importantly, exhibiting antitumorigenic properties when it was overexpressed (Wang et al., 2010; Yao et al., 2015; Huang et al., 2017). However, DNMT3A counteracted the inhibitory effect of miR-101 on the invasive migration of lung cancer cells and also disrupted the miR-101-activated caspase-3 (an apoptotic enzyme) (Yan et al., 2014), which led to the inhibition of NSCLC proliferation (Wang L. et al., 2017). Conversely, Husni et al. observed that almost noninvasive lung adenocarcinomas displayed significant overexpression of DNMT3A and these subtypes had a comparatively better prognosis than others (Weichert and Warth, 2014). Therefore, it was demonstrated that the downexpression of DNMT3A in lung adenocarcinoma might be together with a poor prognosis. Furthermore, Gao et al. (2011) have illustrated that the deletion of DNMT3A promoted the progression of lung cancer in a mouse model. The data uncovered that DNMT3A might act as a TSG, and its suppression might result in the progression of lung cancer. In another study, CD147 has been reported to be closely associated with chemoresistance and aggressiveness, and it stimulates hyaluronan synthesis, thereby affecting the cancer microenvironment (Toole, 2020). Subsequently, Liao et al. (2022) also presented that DNMT3A-associated targeted methylation system downmodulated the CD147 expression level and inhibited NSCLC invasion and metastasis.

In addition to discoveries of roles in lung cancer, DNMT3A has recently attracted considerable attention due to its high expression in the tissues of CRC. In a study on the molecular mechanism of thymine-DNA glycosylase (TDG) treatment for CRC, Miao et al. (2022) found that TDG could bind to and promote the ubiquitination and degradation of DNMT3A, by inhibiting CRC migration and invasion. In the same vein, Zhang et al. noted that targeting the 3′-UTR of DNMT3A mRNA decreased the protein level of DNMT3A and inhibited the proliferation, migration, and invasion of CRC cells (Zhang et al., 2022).

According to the literature, DNMT3A mutations in AML were first described by Ley et al. (2010), and since then several exomes and targeted resequencing studies have identified DNMT3A mutations in AML (Spencer et al., 2017). In addition, the transcription factor CCAAT enhancer-binding protein alpha (CEBPA) controls lineage-specific gene expression and is mutated in a subset of AML (Mannelli et al., 2017). In a significant analysis and discussion on oncogenic role of DNMT3A in AML, Chen et al. (2022) revealed that CEBPA interacted with DNMT3A N terminus (a required structure for normal development and DNA methylation at DNMT3A1-enriched regions) (Gu et al., 2022), preventing DNMT3A from binding DNA and catalyzing CpG methylation. Once cancer-associated mutations in CEBPA, it relieved DNMT3A inhibition and promoted AML. Nonetheless, DNMT3A has some properties that indicate it is also a tumor suppressor in hematologic malignancies. The data from several studies supported this hypothesis owing to the fact that the DNMT3A-deficient model could develop lethal hematologic malignancies after the acquisition of cooperating mutations (Celik et al., 2015; Guryanova et al., 2016), and the overmethylation of CpG islands by DNMT3A may represent a residual trace of a failed attempt to limit proliferation and/or aberrant self-renewal (Miao et al., 2022).

Overall, there seems to be some evidence to indicate that DNMT3A can serve both as an oncogene and as a TSG. The paradoxical effect promoted more researchers to focus on the exploration of DNMT3A, which is conducive to further leaps in tumor-targeted therapy.

### 4.3 DNMT3B in cancer

The DNMT3B gene, located on chromosome 20, has a total protein-coding region of 2,538 bp and encodes a protein of about 95 kDa (Xie et al., 1999). It is hyperexpressed in totipotent embryonic stem cells during the early developmental stages in mesenchymal cells than DNMT3A (Takeshima et al., 2006). Furthermore, there are few reports on DNMT3B in cancer. However, studies have previously reported that DNMT3B was an oncogene that played a crucial role in the progression of various types of cancer, including endometrial cancer (EC) (Gui et al., 2021), lung cancer (Yang et al., 2014), and PCa(Agarwal et al., 2013), and conversely DNMT3B has implicated as a TSG in lymphoma (Hlady et al., 2012).

By using the assays of cell viability, cell cycle, and colony formation, Gui et al. (2021) have been able to identify that DNMT3B was upregulated in EC samples from patients, and promoted EC cell proliferation. This is consistent with the findings of Xiong et al. and Yang et al. (Xiong et al., 2005; Yang L. et al., 2017). Additionally, Ibrahim et al. (2018) found that DNMT3B was highly expressed in colon cancer and was a key link between chronic inflammation and cancer. It was found that DNMT3B mediated epigenetic reprogramming in metastases, innovatively linking the metastatic microenvironment to epigenetic alterations occurring in metastases, which suggested that DNMT3B could be an underlying target for the treatment of metastatic cancer (So et al., 2020). Moreover, Roll et al. (2008) investigated the relationship between the DNMT3B expression levels and methylation levels in BC cell lines. They demonstrated that DNMT3B was significantly elevated in the hypermethylated cell lines. Similarly, DNMT3B mediated the transcriptional repression of the plakoglobin gene through the induction of its promoter hypermethylation, which in turn caused a phenotypic transformation of BC cells (Shafiei et al., 2008). Although there were ample evidences for the cancer-promoting effects of DNMT3B, a study on its role in lymphoma suggested a different perspective. Hlady et al. (2012) deliberately inactivated DNMT3B in T-cells in a mouse model of MYC-induced lymph angiogenesis, leading to increased cell proliferation and accelerated lymphoma development. In the study process, they found that numerous gene promoter was not methylated in DNMT3B^−/-^ pretumor thymocytes, which implicated that DNMT3B maintained cytosine methylation in cancer. In short, the findings identified that DNMT3B was a lymphopoietic potentiator that contributed to a potential target for anticancer therapies.

Certain genes have a double-edged role in cancer, as is the case with DNMT3B, thus it is worth exploring the further mechanism of DNMT3B’s role in cancer.

### 4.4 DNMT intermediates in cancer

Mechanistically, the review of the extensive literature indicated that the various subtypes of DNMT not only functioned independently but were also closely interconnected and collectively influenced cancer progression. It has been reported that DNMT3B can act as an important cofactor, usually in conjunction with other DNMTs. For instance, Rhee et al. (2002) demonstrated that DNMT1 and DNMT3B collectively maintained virtually all methylation in HCT116 cells (a cell from CRC), including the normal methylation of repeated sequences and the silencing-associated methylation genes like insulin-like growth factor 2 (IGF2), TIMP metallopeptidase inhibitor 3 (TIMP3), and p16. In addition, the results also showed a more significant demethylation effect of the double mutant clone compared to the single DNMT1 and DNMT3B mutants. Apart from that, recent studies have also revealed that the diverse functions of C-X-C Motif Chemokine Ligand 11 (CXCL11) included restraining angiogenesis, disrupting the proliferation of different cell types, playing a role in cancer invasion, and upregulating adhesion properties (Gao and Zhang, 2021). Based on that, a study has shown that DNMT3B could comediate CXCL11 with DNMT1 to suppress BC malignant phenotype (Li et al., 2022). By coincidence, in DU145 cell (a cell line of PCa), the related gene was regulated epigenetically by DNMT1 and DNMT3B *via* targeted hypermethylation, and that consequent gene overexpression promoted prostate carcinogenesis (Zhu et al., 2021). In another research, the findings have shown here that DNMT1 and DNMT3A functionally cooperated in *de novo* methylation of DNA, for the reason that a five-fold stimulation of methylation activity is found if both enzymes are present (Fatemi et al., 2002). Additionally, it has been demonstrated that the active features of BC-associated fibroblasts cells, major components of the tumor microenvironment, could be normalized through drug targeting of DNMT1/DNMT3A and the consequent modulation in gene methylation (Al-Kharashi et al., 2021). Apart from that, in multivariate logistic regression, Ma et al. (2018) revealed that the obvious association between DNMT1 or DNMT3A and hypermethylation status persisted after adjusting for the clinicopathological characteristics. It was subsequently concluded that the overexpression of DNMT1 and DNMT3A in cancer was associated with aggressive behavior and hypermethylation status of pituitary adenomas.

Considering all these evidences, it seems that DNMTs are inextricably linked to each other and they act as cofactors with each other in cancer. Therefore, in the future, multipoint strikes against the network of DNMTs will become a new idea for anticancer therapy.

## 5 Regulated mechanism of DNMTs in the pathogenesis of cancer

Exploring the function and signaling pathways involved in genes is often a practical way of exploring the molecular mechanisms of disease. It must be accepted that the occurrence, progression, metastasis, invasion, and other processes of cancer tumor touch upon complex molecular mechanisms, while the current comprehension of them is incomplete, which induces a low recovery rate of cancer. Therefore, the researchers focused on the role of DNMTs on cancer at a molecular level and found that the mitogen-activated protein kinase (MAPK) signaling pathway, the ubiquitous, growth factor-regulated phosphoinositide 3-kinase (PI3K)/AKT signaling pathway, as well as the Wnt/β-catenin signaling pathway, were activated in the development of cancer.

**FIGURE 2:**
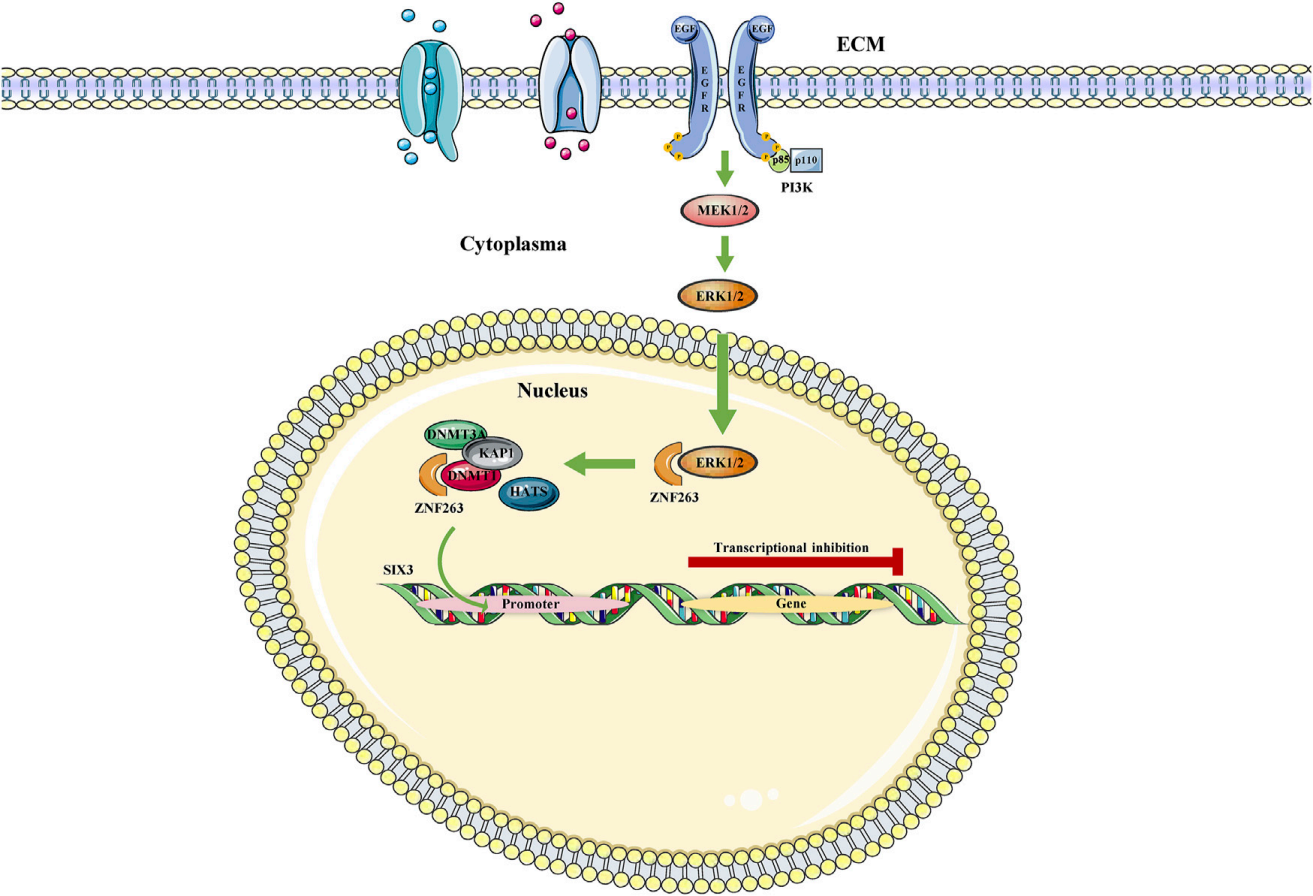
MAPK/ZNF363/DNMTs/SIX3 signaling pathway. The MAPK signaling pathway represses SIX3 transcription by regulating ZNF263, a repressor gene of Six3, in the nucleus.

The MAPK signaling pathway is a key signaling system in eukaryotic cells mediating the intracellular response to extracellular signals and transducing extracellular signals through a tertiary kinase cascade. It has been shown to regulate a variety of cellular activities participated in cancer progression, including proliferation, apoptosis, and immune escape (Peluso et al., 2019). In a study of Cui et al., Zinc finger protein 263 (ZNF263) acts as a functional endoplasmic reticulum stress (ERS)-related tumor activator, increasing cancer chemoresistance by activating ERS-related autophagy (Cui et al., 2020). Under the study background, Yu et al. (Yu Z. et al., 2020) found that the MAPK signaling pathway can be activated by EGFR and subsequently inhibits ZNF263 ubiquitination. When ZNF263 protein was enriched, it continued to recruit DNMT1/DNMT3A/HMT, induced transcriptional silencing of the SIX homeobox 3 (SIX3) promoter, and triggered or enhanced the oncogenic activity of glioblastoma (Figure 2). Moreover, a study of PC also revealed that the activation of MAPK signaling pathway in pancreatic epithelial cells mediated the upregulation of DNMT3A and DNMT3B, which further induced aberrant hypermethylation of TSGs, thereby leading to PC development (Jin et al., 2018). As well-known, PCa is a heterogeneous tumor that commonly occurs among males worldwide. In addition, it has been found that Neuropeptide Y1 receptor (NPY1R) promoted proliferation, vascularization, and stimulate migration in tumor (Dawoud et al., 2021). Most recently, a study showed that DNMT1/DNMT3 (A/B) promoted the methylation of the NPY1R promoter, which downregulated NPY1R expression to activate the MAPK signaling pathway and worsen PCa (Li X. et al., 2020).

**FIGURE 3:**
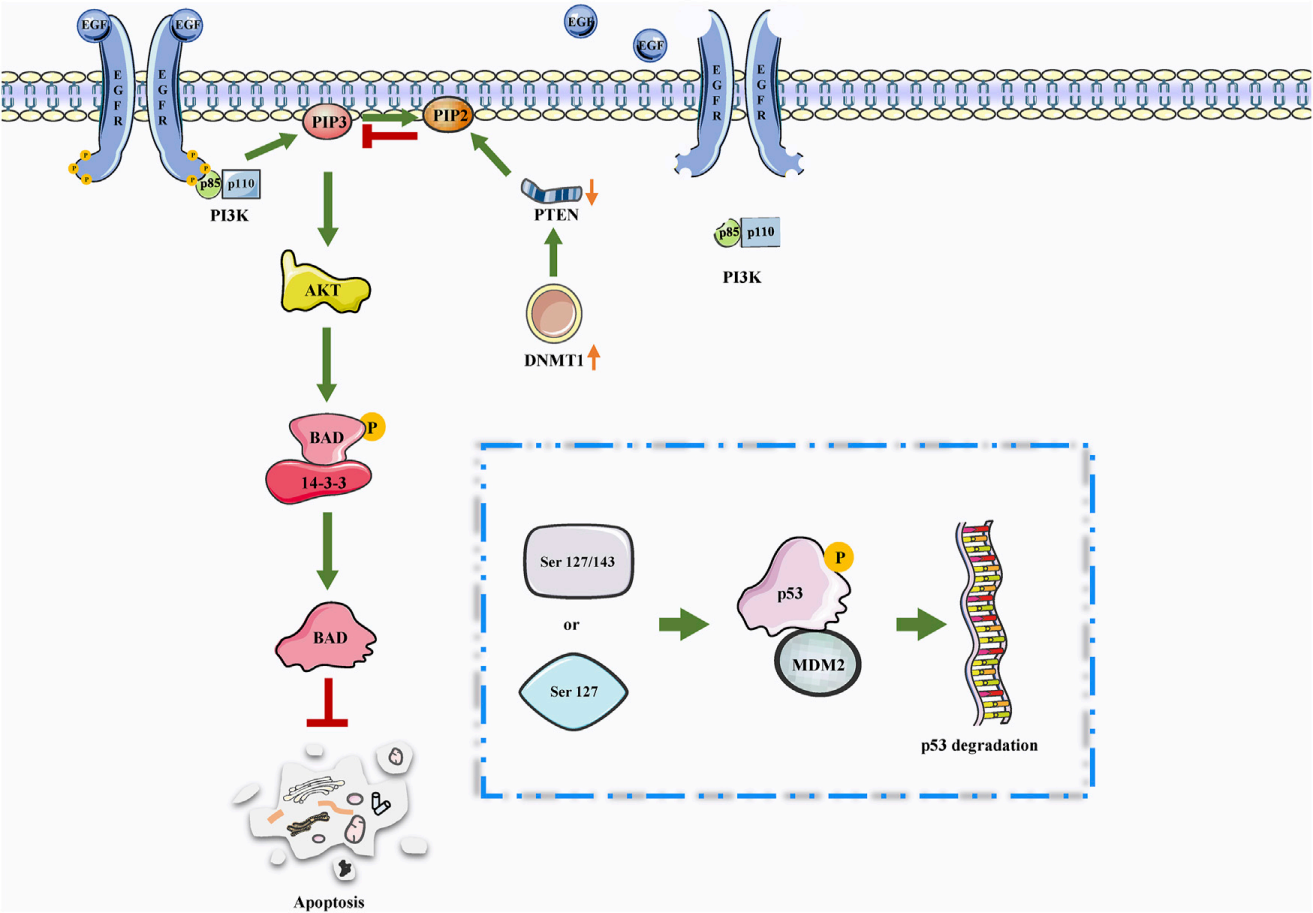
DNMTs may be involved in PTEN/PI3K/AKT. The PTEN/PI3K/AKT signaling pathway is involved in the regulation of apoptosis. Interestingly, the high expression of DNMTs leads to a decrease in PTEN expression. Therefore, DNMTs may further inhibit apoptosis through this pathway.

The PI3K/AKT signaling pathway is the most frequently activated pathway in cancers and works for disconnecting the control of cell growth, survival, and metabolism from exogenous growth stimuli (Lawrence et al., 2014). The genes that make up this pathway have been extensively studied and found to be usually activated in human cancer (Alzahrani, 2019). Some studies have revealed that thioredoxin-interacting protein (TXNIP) is a thioredoxin-binding protein that can inhibit cell proliferation and induce apoptosis, acting as a tumor suppressor in some cancers (Chen et al., 2020). Based on this conclusion, a functional experiment revealed that DNMT1 and DNMT3A upregulation was involved in TXNIP hyperexpression in high glucose-stimulated RSC96 cells. In this process, the inhibition of the PI3K/AKT signaling pathway mediated DNMT1 and DNMT3A overexpression, which caused cell autophagy and apoptosis in Shewan cells of diabetic peripheral neuropathy (DPN) (Zhang et al., 2021). Additionally, PTEN is a classic TSG that downregulates the PI3K/AKT signal pathway, thereby negatively regulating the signaling pathway and inhibiting cancer development (Bazzichetto et al., 2019). Studies have demonstrated that DNMT3A affected the development of lung cancer cells (A549 cells) *via* the PTEN/PI3K/AKT signaling pathway (Wang L. et al., 2017). Furthermore, angiogenesis is a major feature in the development of malignant cancers, resulting in poorly perfused cancers and promoting their aggressiveness. In a recent study on BC, the results have demonstrated that the overexpression of DNMT1 activated the PI3K/AKT signaling pathway, thereby increasing BC angiogenesis (Figure 3).

The Wnt/β-catenin signaling pathway is a common class of signaling pathways in cancer and its activation endows cancer cells with sustained self-renewing growth properties and is associated with therapy resistance (Bugter et al., 2021). In an experiment of Carbajo–García MC et al., the findings have demonstrated that in human uterine leiomyoma primary (HULP) cells, aberrant overexpression of DNMT1 induced abnormal DNA methylation and subsequently activated the Wnt/β-catenin signaling pathway, contributing to cell proliferation and extracellular matrix (ECM) formation (Carbajo-Garcia et al., 2021). In addition, a significant study on the mechanism of treatment for mantle cell lymphoma (MCL) indicated that the inactivation of the Wnt/β-catenin signaling pathway and downregulation of DNMT1 synergistically suppressed MCL, and these results could guide the dosing in the clinical treatment of MCL (Li et al., 2017).

Overall, the DNMTs joined the regulation of many cellular signaling pathways and played the important role in the occurrence, development, and clinical manifestations of various cancers, suggesting DNMTs as a potential target for cancer therapy.

## 6 Potential clinical significance of DNMTs in inflammation and cancer

Cancer is difficult of being detected in the early stage, which leads to almost all cancer patients to miss the best time for surgical treatment when they are diagnosed, causing a low survival rate (Siegel et al., 2020). In recent years, increasing emerging markers provided a better reference to the diagnosis and cure of cancer, and sensitive molecular biomarkers could be of help to identify cancer origin, differentiate precancerous lesions from cancer, and define tumor malignancy, which were helpful for the early diagnosis and treatment of cancer. It is well-known that an ideal biomarker should meet various criteria such as easy accessibility, high sensitivity (allowing early diagnosis), and timely changes in response to treatment and disease progression. Importantly, its detection value should have a quite high correlation with the pathological features and clinical parameters of cancer, such as high Gleason score, advanced cancer stage, positive lymph node status, and incomplete tumor resection (Skovgaard et al., 2016). The relevant research reported that DNMTs expression level was significantly associated with intrusive depth, metastasis, prognosis, and drug resistance of cancer (McMullen et al., 2020; Zhang et al., 2020; Nagaraju et al., 2021). Therefore, this section summed up the reports on potential clinical significance of DNMTs in cancer.

A trial on gemcitabine resistance in AML showed that among 122 patients in a combined phase I and phase II cohorts, cluster R (resistant) patients characterized by downregulated DNMT1 and upregulated DNMT3A/DNMT3B were resistant to gemcitabine, with composite complete response (CRc) observed in 0/27 (0%) patients and 28/95 (29%) patients for cluster S (sensitive) (*p* = 0.0005). In addition, further analysis using univariate Cox regression showed that the overexpression of DNMT3B was related to worse overall survival (OS) (hazard ratio (HR) = 1.26, 95% CI 1.07–1.49, *p* = 0.005) (Chung et al., 2019). Apart from AML, similar results have been also found in PCa. The comparison between the samples from drugged or unmedicated PCa patients showed that DNMT3B [9/39 (23.0%) vs. 24/51 (47.1%) (*p* = 0.034)] and DNMT3A [21/39 (53.9%) vs. 41/51 (80.4%) (*p* = 0.014)] were upregulated in the treatment group, while DNMT1 expression did not change [25/39 (64.1%) vs. 37/51 (72.5%) (*p* = 0.530)]. Moreover, the expression level of DNMT also changed with tumor differentiation, from 52.9% to 68.4% (*p* = 0.543) for DNMT1, 29.4%–68.4% (*p* = 0.045) for DNMT3A, and 5.9%–31.6% (*p* = 0.128) for DNMT3B when changing from Gleason less than or equal to six to Gleason 7, of which only DNMT3A expression differences were significant, with a Gleason score of 8–10, DNMT1 (*p* = 0.252) and DNMT3A (*p* = 0.016) expression was 100% and DNMT3B expression was 66.7% (*p* = 0.033) (Gravina et al., 2011). Together, these studies statistically proved the potential of DNMTs as diagnostic biomarker.

In addition to the statistical data analysis, some studies have validated the effect of DNMTs on tumors through *in vivo* trials. In a recent study, Pathania R et al. (Wang et al., 2018) built mammary gland-specific conditional *Dnmt1*-knockout mice to identify the role of DNMT1 in regulating mammary stem/progenitor cells. After crossing *Dnmt1* ^Δ/Δ^ mice with MMTV-Neu-Tg mice, which mimic human luminal progenitor (LP) cell of origin, and C3 (1)-SV40-Tg, which mimic human basal triple-negative BC, results showed that *Dnmt1*-knockout remarkably suppressed cancer incidence, tumor size, and tumor sphere formation capacity. Additionally, another study supports this finding. The result was initiated using the subcutaneous injection of mouse PCa cells transfected with HSV1tk-GFP-luciferase (SFG-nTGL) reporter gene expression vector into the right flank region of C57BL/6 mice. Subsequently, following intraperitoneal injections with the specific anti-TGF-β neutralizing antibody 1D11 or control antibody 13C4, the mice were randomly assigned to three groups. The results showed that DNMTs were significantly lower in those mice regularly injected with the 1D11 antibody than in other groups, and tumor growth was significantly inhibited (Zhang et al., 2011).

Taken together, DNMTs are of clinical importance in cancer diagnosis. Nonetheless, the mechanisms involved remain unclear, in which case more in-depth exploration and more complete trials are necessary for elucidating the role of DNMTs in cancer.

## 7 Future outlook

With more and more in-depth studies, the vital role of DNMTs in cancer is being concerned and understood. Recent studies have indicated that the expression level of DNMTs were abnormally elevated in BC (Zhu et al., 2022), EC (Gui et al., 2021), and GC (Lu et al., 2021), and therefore promoted carcinogenesis process and subsequent metastasis and invasion. Therefore, targeting DNMTs to improve disease progression is supposed to be a novel and effective strategy to treat cancer. In this section, we will fully look forward to the full DNMTs, based on the novel technologies and latest research results in life sciences.

Recently, the improvement of surgery, radiotherapy, and chemotherapy has made a major breakthrough in cancer treatment. However, the side effects brought by the abovementioned treatment and the drug resistance of anticancer drugs have not improved the life expectancy and the survival rate (Jung et al., 2019; Codolo et al., 2022). It is well-known that the occurrence of cancer is due to the changes in the genome and epigenome, mainly manifested in the activation of oncogenes or the inactivation of tumor suppressor genes (Khan et al., 2016). Therefore, researchers hope to improve the therapeutic effect and prognosis of cancer at the gene level, that is, from the perspective of the fundamental mechanism of cancer pathogenesis. Researchers have previously induced endogenous siRNA process pathways for RNA interference to trigger post-transcriptional gene silencing, or used DNA targeted homologous recombination to achieve gene targeting (including gene knockout or knock-in) (Yi and Liu, 2011). Fortunately, with the emergence of the clustered regularly interspaced short palindromic repeats (CRISPR)/CRISPR-associated nuclease 9 (Cas9) system which acted as an RNA-guided genome editing tool, therapeutic gene editing is becoming a viable biomedical tool and was first tested in a person in 2016 (Cyranoski, 2016; Luther et al., 2018). The modular organization of this tool allows it to be used not only for DNA modifications but also for introducing epigenetic modifications in DNA, like methylation and demethylation (Jeffries, 2018; Ignatova et al., 2019). Therefore, it is easy for us to associate that editing DNMTs by CRISPR/Cas9 might be an effective and prospected strategy against cancer. Moreover, previous results have demonstrated that DNMT1 acts as a promoter of lung cancer, and the safety and feasibility of CRISPR/Cas9 gene-editing approaches in NSCLC have been verified (Lu et al., 2020). Thus, the knockdown of DNMT1 by using the CRISPR/Cas9 system would be a new strategy to repress proliferation, migration, and invasion of lung cancer cells. Subsequently, for the CRISPR/Cas9 system to efficiently engage in epigenetic alterations, researchers have noticed an inactivated Cas9 protein variant, leading to the discovery of the dCas9 system, a novel dCas9-mediated editing system (Jinek et al., 2012). In one of the designs, DNMT3A is fused with dCas9 resulting in the dCas9-DNMT3A complex, which causes the suppression of genes by increasing methylation in the promoter region through the enzymatic action of DNMT3A (McDonald et al., 2016). Hence, it was further speculated that designing a CRISPR-dCas9-DNMT3A system to target the downstream genes of DNMT3A could achieve the suppression of tumorigenesis. In several studies, the dCas9-Effector system has been utilized to understand or identify the role of the gene in oncogenesis or tumorigenesis and to identify the epigenetic targets for cancer therapy (Saunderson et al., 2017; Wu et al., 2019). Thus, DNMTs, as an epigenetic modifier, are expected to apply to epigenome editing therapies. In sum, CRISPR/Cas9 addresses or corrects epigenetic alterations through DNMTs, an approach that will open up new frontiers for cancer therapy in the future.

Lately, the interlink between epigenetic mechanisms in cancer has aroused widespread concern, especially DNA methylation and histone deacetylation. A significant correlation between epigenetic mechanisms DNA methylation and histone deacetylation has been reported by Nan et al., which showed that two most studied epigenetic mechanisms, that is, DNA methylation and histone modification, can be linked by Methyl-CpG binding protein 2 (MeCP2) (Nan et al., 1998). Furthermore, this growing body of evidence has demonstrated that histone deacetylases (HDACs) are linked to the initiation and/or maintenance of repression for DNA hypermethylated genes, and studies have revealed that the simultaneous targeting of both DNA methylation and histone deacetylation leads to additive or synergistic effects to reactivate the aberrantly silenced genes (Arrowsmith et al., 2012). Meanwhile, it has been observed that in the case of histone methylation, methylation of histones H3 and H4 is a very common feature of cancer cells (Tan et al., 2022). Notably, it was reported that DNMT demethylating agents and histone deacetylase inhibitors could be combined to exert antitumor effects (Zahnow et al., 2016; Myasoedova et al., 2019). In summary, we hypothesized that there was a relationship between DNMT methylation and histone acetylation, and the effects of both on cancer deserve further investigation.

In conclusion, DNMTs are not only the novel connecting point of basic life science and clinical medicine research but also can be applied in combination with many other Frontier research results, representing a promising research direction and providing new ideas for clinical cancer treatment.

## 8 Conclusion

Recent evidence has suggested a close link between DNMTs and the tumors’ pathogenesis, throughout tumor invasion, proliferation, metastasis, diagnosis, and prognosis. DNMTs have been found to be aberrantly expressed in a variety of malignancies, including BC, PCa, and CRC, in which the regulation of lymphoma shows a dual nature. Also, it has been shown that DNMTs regulate tumors both *in vivo* and *in vitro* and are associated with OS and distant metastasis of cancer. Meanwhile, DNMTs were implicated in the regulation of several signaling pathways in cancer cells. For instance, the alpha 7 nicotinic acetylcholine receptor (alpha7nAChR) and MAPK signaling pathways induce aberrant hypermethylation of TSGs by regulating DNMT in pancreatic epithelial cells. Nevertheless, up to now, reported studies are only the tip of the iceberg in the exploration of DNMTs. In conclusion, more high-quality clinical trials and intensive exploration of mechanisms will be the future research priorities and directions for DNMT.

## Glossary

DNMTs: DNA methyltransferases
BC: Breast cancer
PCaProstate cancerSAM: Prostate cancerSAMS-adenosylmethionine
C: Cytosine
CH3: Methyl
DALYs: Disability-adjusted life years
MDSs: Myelodysplastic syndromes
AML: Acute myeloid leukemia
PTEN: Phosphatase and tensin homolog
MTUS1: Microtubule-associated tumor suppressor 1
NSCLC: Non-small-cell lung cancer
hMLH1: Human MutL Homolog 1
hMSH2: Human MutS Homolog 2
PDAC: Pancreatic ductal adenocarcinoma
GC: Gastric cancer
TAMs: Tumor-associated macrophages
MHC: Major histocompatibility complex
DMTi: DNA methyltransferase inhibitor
GNAO1: G Protein Subunit Alpha O1
HCC: Hepatocellular carcinoma
CRC: Colorectal cancer
TDG: Thymine-DNA glycosylase
CEBPA: CCAAT enhancer-binding protein alpha
EC: Endometrial cancer
CXCL11: C-X-C Motif Chemokine Ligand 11
IGF2: Insulin-like growth factor 2
TIMP3: TIMP metallopeptidase inhibitor 3
MAPK: Mitogen-activated protein kinase
PI3K: Phosphoinositide 3-kinase
ZNF263: Zinc finger protein 263
PC: Pancreatic cancer
NPY1R: Neuropeptide Y Receptor Y1
TXNIP: Thioredoxin-interacting protein
DPN: Diabetic peripheral neuropathy
TSG: Tumor suppressor gene
HULP: Human uterine leiomyoma primary
ECM: Extracellular matrix
MCL: Mantle cell lymphoma
CRc: Composite complete response
OS: Overall survival
HR: Hazard ratio
CRISPR: Clustered regularly interspaced short palindromic repeats
Cas9: CRISPR-associated nuclease 9
MeCP2: Methyl-CpG binding protein 2
HDAC: Histone deacetylases
alpha7nAChR: Alpha 7 nicotinic acetylcholine receptor
